# Influence of the Composition and Curing Time on Mechanical Properties of Fluidized Bed Combustion Fly Ash-Based Geopolymer

**DOI:** 10.3390/polym13152527

**Published:** 2021-07-30

**Authors:** Natalia Wielgus, Jan Kubica, Marcin Górski

**Affiliations:** Department of Structural Engineering, Faculty of Civil Engineering, The Silesian University of Technology, 44-100 Gliwice, Poland; natalia.paszek@polsl.pl (N.W.); marcin.gorski@polsl.pl (M.G.)

**Keywords:** geopolymer, FBC fly ash, metakaolin, CRT glass, curing regime, flexural and compressive strength

## Abstract

This paper presents novel research on a fluidized bed combustion (FBC) fly ash-based geopolymer as a contribution to the problem of FBC fly ash disposal, and a proposal for a new geopolymer composition—an environmentally friendly material that is possible to use in construction. Geopolymer samples of various composition (containing FBC fly ash as the main raw material, metakaolin and CRT glass as additional components, and sodium silicate and sodium hydroxide as activators) were subjected to flexural and compressive strength tests. An investigation on the effect of the demolding time was carried out on one selected mixture. The test showed that both the composition and the demolding time have a decisive influence on the basic mechanical properties. A mixture containing FBC fly ash to metakaolin in a mass ratio of 3:1, removed from the mold after 14 days, was found to be the best in terms of the mechanical parameters expected from a material that could be used in construction, e.g., for the production of precast elements. According to the results obtained, FBC fly ash is a promising and environmentally friendly raw material for the production of geopolymer, with good mechanical properties and low density. Moreover, a high compressive strength can be obtained by curing the geopolymer at ambient temperature.

## 1. Introduction

Fluidized bed combustion (FBC) technology was introduced to the market in the 1970s as a cleaner method of energy production. The fluidization process starts at the moment when the flow of air introduced inside the boiler suspends a bed of inert material (granular, solid particles). The increasing velocity of the gas stream leads to the suspension of the particles inside the bed. Then, the fuel—and optionally the sorbent—may be injected into the boiler [1]. Fluidized bed combustion allows for the utilization of low-grade solid fuels of different quality, moisture content and composition [2]. The FBC technology process results in three main products: flue gas desulfurization gypsum, and two types of FBC ashes (bottom, also called as bed ash and fly or filter ash) of which the composition is variable in chemical and phase aspects. That feature, together with usually high CaO and SO_3_ content and low pozzolanic activity, as well as increased water demand, limits the possibility of the application of FBC ash in some branches such as the concrete industry [3,4,5] or civil engineering. Nevertheless, such wastes can find application in road bases, stabilization of soils, production of synthetic aggregates, structural fill, mine backfilling, as a filler in polymer composites etc. [2,6]. The other approach is to change or valorize the composition of FBC ash, or to increase its pozzolanic activity to enable its further application [7,8,9]. Regardless of some limitations, there are plenty of reported studies on the application of FBC ash in construction branches [2], such as a replacement of Portland cement in pavement-grade concrete [10,11]. Concrete containing FBC fly ash admixture is able to achieve good compressive strength, proper slump, air-void factor, acceptable drying shrinkage performance, as well as plastic and hardened state air contents, and is characterized by longer setting, better resistance to chloride ion penetration, and enhanced corrosion durability by increased resistance to sulphate attack [10]. Some studies report enhancement of compressive strength after addition of FBC ash [11]. Tests have also been performed on autoclaved aerated concrete (AAC) with FBC ash, which shows good compressive strength and volume stability [12]. The potentially negative effects caused by high water demand can be alleviated by using specific additives [11,13]. Frequently reported excessive expansion of binders containing FBC ash (caused mainly by the SO_3_ content) can be controlled by the grinding of FBC ash or by the addition of aggregates [14]. The next study shows compressive strength improvement while using small amounts of FBC ash in modified high volume, and low calcium fly ash [15]. The other studies describe high durability, decreased permeability and faster hydration of concretes and mortars incorporating FBC ash [2]. Although there are numerous examples of utilization of FBC ash, there are usually limitations concerning the amount which can be safely applied, especially in concrete branches [2,4,10,11,15,16,17,18,19,20]. Therefore, the new possibilities of utilization of such type of wastes are still demanded.

One of the relatively new branches of science which give the possibility of utilization of FBC ash are geopolymers—alkali aluminosilicate binders being a product of aluminosilicate materials activated by alkali silicates [21]. Geopolymers are treated as an innovative material, providing plenty of opportunities for application, among the others in the building industry, as an environmentally friendly alternative for concrete [22]. Geopolymers allow for some savings in greenhouse gas emissions [23,24,25], enable recycling of wastes, (often hazardous ones, such as crushed cathode ray tube (CRT) glass, containing lead which would have to be recovered e.g., by a chemical–electrochemical process [26]) [23,27,28], can achieve comparable or even better strength values than ordinary Portland cement (OPC) concrete [29,30]. Moreover they show properties such as fire and heat resistance [31,32,33], acid, chloride, and corrosion resistance [33,34], and the ability to immobilize toxic metals [35]. Geopolymers can also be combined with other ecological resources such as natural fibers (sisal, jute etc.), becoming environmental friendly composites of enhanced mechanical behavior [36]. There are various possible aluminosilicate sources for geopolymers, such as blast furnace slag, metakaolin, or fly ash [21,36]. Many studies also describe various mine wastes-based geopolymers [37]. The application of FBC ash in geopolymers is a promising way to utilize that type of waste. Numerous successful attempts of producing geopolymers based on FBC ash have been reported [2]. In [38], authors reported that both SEM photomicrographs and the FTIR spectra prove the conversion of circulating fluidized bed combustion bottom ash to geopolymer. FBC ash-based geopolymers are able to achieve high values of basic mechanical properties such as compressive strength, gaining from 40 up to over 70 MPa [38,39,40,41]. However, many studies report smaller values of compressive strengths, especially when FBC ash is the only raw material [42,43,44,45,46]. Some studies describe the possibilities of enhancement of the reactivity of FBC ash leading to a higher strength, for instance by an alkali fusion pretreatment [45,47] or high-shear granulation [48] of raw material.

What is more, FBC bottom ash-based geopolymer shows superior thermal stability in comparison to OPC concrete. The exposure to high temperatures (up to 1050 °C) may even enhance the material’s compressive strength, which gives the possibility of application of FBC bottom ash-based geopolymer in places where thermal stability is crucial [38]. The increment of strength after exposure to thermal treatment probably occurs due to the viscous sintering and better completion of the geopolymerization process [38,41]. FBC fly ash is characterized by smaller shrinkage than OPC concrete when exposed to high temperatures [41]. The FBC fly ash geopolymer, except for its excellent heat resistance, shows resistance to acidic solutions, losing nearly five times lower strength compared to OPC concrete. It also shows lower water absorption than OPC concrete [41]. The possible ettringite formation (which may lead to the internal expansion and deterioration of hardened material) can be controlled by the proper NaOH concentration and Si/Al ratio. According to previous research, the high NaOH concentration (15 mol/L) reduces the ettringite formation and increases the compressive strength of geopolymer [44]. FBC ash can also be used as a raw material for the preparation of foam geopolymer [49] or lightweight geopolymer aggregates [48].

Some studies report preparation of geopolymers using FBC ash as the only raw material [38,39,42,43,44,45,46,49]. However, it is also popular to blend FBC ash with a variety of additional materials such as kaolinitic clay, pulverized coal combustion fly ash, metakaolin, cement, or blast furnace slag [39,40,41,43,46,47,48,50,51].

Notwithstanding the numerous existing studies describing the use of FBC ashes in geopolymers, there are still many possible compositions to discover, especially considering the variability of FBC ash parameters from different sources [2]. Moreover, in the case of geopolymers, even small changes in composition can have a crucial impact on the characteristics of hardened material [52]. This paper presents the study on the determination of the optimal mixture and hardening regime of geopolymer based on FBC fly ash from one of the Polish power plants. A geopolymer with good mechanical properties would be a valuable contribution to the problem of disposal of a specific type of waste, e.g., in the broadly understood concrete prefabrication, and also including production of precast load-bearing structural elements, such as reinforced girders or plates. The importance of using local wastes in geopolymers is often emphasized by researchers [37]. In addition, most of the studies to date describe a material cured at elevated temperatures [42,43,44,45,49,50,53,54], while the current work presents the FBC fly ash geopolymer produced at ambient temperature, which is better for both ecological and economic reasons. The previous investigations were frequently based on geopolymers activated with a high NaOH concentration (10–15 mol/L) [40,41,42,43,44,46] while the following paper presents a good mechanical performance of a geopolymer activated with NaOH of a lower concertation (6 mol/L). Low concentration of an activator is another factor decreasing production costs and negative environmental impact. The behavior of the geopolymer based on FBC fly ash after curing at elevated temperatures was investigated and presented in the authors’ previous research [55].

The main goal of the paper was to find an optimal mixture composition for local, Polish FBC fly ash-based geopolymer in terms of its suitability for use in construction prefabrication. A few mixtures of different compositions (containing FBC fly ash and additional materials) were investigated. In the next step of the research, an optimal time of demolding of samples was determined on one chosen mixture. The compressive and flexural strengths (as well as the lack of external deterioration caused by shrinkage) of hardened geopolymer were the main indicators of the quality of the chosen mixture and the accuracy of the demolding time. Compressive and flexural strengths (related to the crack resistance, both connected to shrinkage and tensile stresses occurring in the material, generated by internal forces in a structural member) are the basic mechanical parameters from the point of view of the usefulness of the geopolymer for possible applications in construction, particularly in structural precast elements. Therefore, the presented research focuses primarily on these two mechanical parameters.

## 2. Materials and Methods

Circulating fluidized bed combustion (FBC) fly ash from the Polish power plant was used as the main raw material in all mixtures. Material was a mixture of a fly ash (being a by-product of hard coal combustion in fluidized technology) and solid products coming from desulphurization of fumes using dry methods. Material was produced in a circulating fluidized bed at a temperature around 800 °C. The special sorbent (calcium carbonate) in the form of limestone sand was introduced to the system except for hard coal. FBC fly ash was delivered by Tauron Polska Energia SA Company.

Metakaolin (Astra MK 40), used as supplementary raw material in two mixtures, is a commercial product supplied by the company Astra Technologia Betonu.

**Figure 1:**
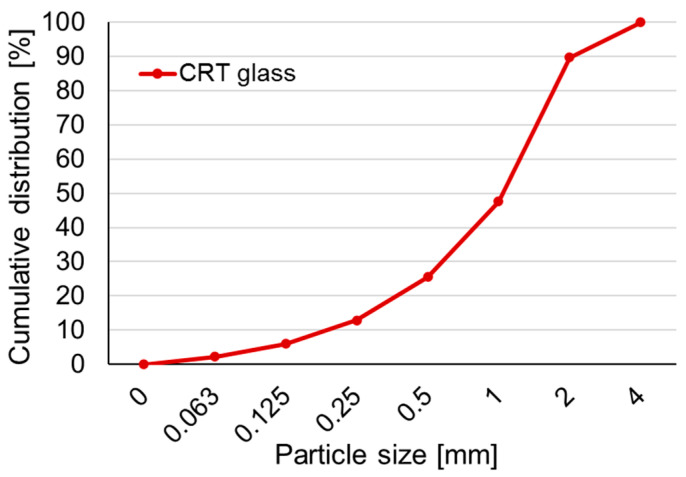
The particle size distribution of CRT glass.

Crushed cathode ray tube (CRT) glass was used as an aggregate in one mixture. Material came from old, discarded computer monitors and television sets based on cathode ray tubes. Glass was collected, separated, crushed and delivered by the Thornmann Recycling company. The material consisted of all types of CRT glass mixed together; fractions less than 4 mm were applied in the test. Glass was not subjected to any treatment before use. The curve presenting the particle size distribution of CRT glass is shown in Figure 1. Table 1 contains the chemical composition of FBC fly ash, metakaolin, and CRT glass.

The mixture of commercial sodium silicate solution and sodium hydroxide was used as an activator in all mixtures. The producer provided the following characterization of sodium silicate solution: ratio of SiO_2_ to Na_2_O between 2.4 and 2.6. The minimum content of oxides (SiO_2_ and Na_2_O) equals 39%. Sodium hydroxide solution was prepared using sodium hydroxide pellets and demineralized water for a minimum of 24 h before the preparation of samples. Sodium hydroxide was dissolved in water in such an amount to prepare a solution of concentration 6 or 10 mol/L.

Four mixtures of variable composition were prepared. Table 2 contains detailed information about the composition of each mixture. The first mixture was composed of FBC fly ash (FBC FA) only, the second one of FBC fly ash and crushed CRT glass (in the form of aggregate), the third one contained FBC fly ash and metakaolin in mass ratio 3:1, while the fourth mixture contained FBC fly ash and metakaolin in mass ratio 4:1. All mixtures were activated with the use of sodium silicate and sodium hydroxide. The concentration of sodium hydroxide is given in the table for each mixture separately.

## 3. Tests Procedure

All mixtures were prepared according to the same procedure. All dry ingredients were firstly mixed together and then blended with activators (which were previously stirred for 5 min with the use of a magnetic stirrer). The mixture was placed in prismatic forms of dimensions 40 mm × 40 mm × 160 mm, compacted, covered tightly and cured at an ambient temperature (~20 °C) in the laboratory for the complete curing period. Geopolymer samples subjected to the test described in point 4.1 of the paper were demolded just before the test. Samples used for the determination of the influence of curing regime on mechanical behavior (point 4.3 of the paper) were demolded after 7, 14 or 28 days of curing.

Before the strength tests, each test specimen was weighed and measured. The density of geopolymer was calculated by dividing mass by the volume of each sample. All samples were subjected to flexural and compressive tests according to the requirements given in the European standard EN 196-1:2018 [56]. In the first step, to determine the flexural strength, each prism was subjected to the three-point bending test. The compressive strength test was performed on halves of samples broken during the flexural strength test. Tests were carried out on the machine Controls^®^ model 65-L27C12, serial no. 12020060 (Controls, Milan, Italy).

## 4. Results and Discussion

The compressive and flexural strength test results are presented on bar charts. The value given above each bar represents the average value from the results of the following series of samples. The black segment in the upper part of each bar represents the lowest and the highest result in each series.

### 4.1. The Influence of the Composition of a Mixture on Mechanical Behavior—A Test Done after 7 Days of Curing

**Figure 2:**
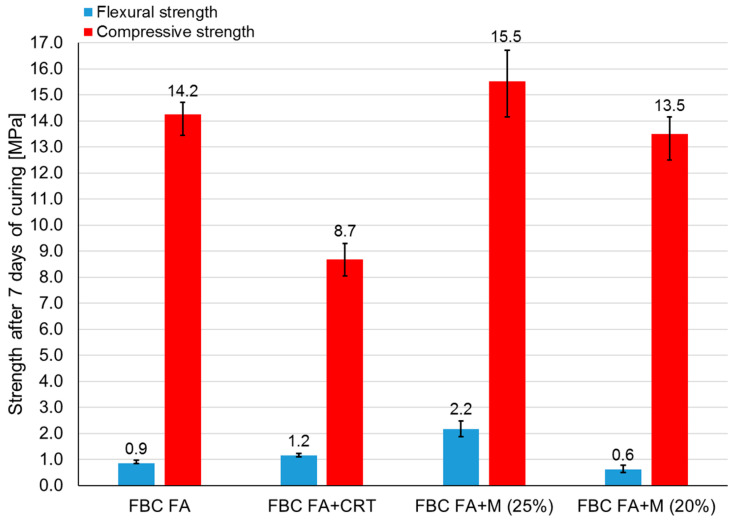
Flexural and compressive strength of geopolymer samples made of different mixtures.

Figure 2 shows the flexural and compressive strength of samples prepared from different mixtures, respectively. The test was carried out after 7 days of curing at the ambient temperature (~20 °C). Six prismatic samples were tested in each series. Consequently, six samples were subjected to a flexural strength test and 12 samples to a compressive strength test. All samples achieved a rather low flexural strength. Samples made of mixture FBC FA + M (25%) had a considerably higher flexural strength than samples made of the remaining mixtures (over two times higher). Compressive strength values were close to each other in the case of all mixtures except those made of mixture FBC FA + CRT, which achieved significantly lower results. Table 3 presents the standard deviations and coefficient variations of all results.

### 4.2. The Influence of the Composition of a Mixture on Mechanical Behavior—A Test Done after 28 Days of Curing

**Figure 3:**
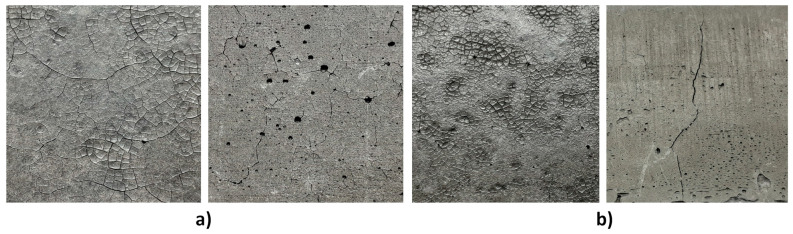
Cracked surface of samples after 28 days (**a**) upper and side (left-right) surface of the sample made of mixture FBC FA + CRT; (**b**) upper and side (left-right) surface of the sample made of mixture FBC FA.

**Figure 4:**
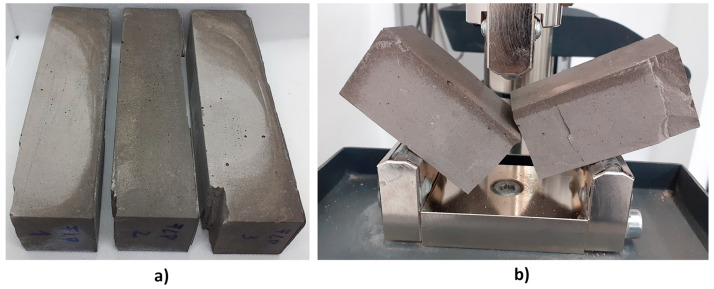
Samples made of mixture FBC FA + M (25%) demolded after 7 days; picture taken after 28 days of curing (**a**) before the strength tests; (**b**) during the three-point bending test.

Samples subjected to the 28 day strength test were demolded after 7 days. Only those made of mixtures FBC FA, FBC FA + CRT and FBC FA + M (25%) were examined. After 28 days, the net of shallow cracks most probably caused by the shrinkage (Figure 3) affected the surface of samples made of mixtures FBC FA and FBC FA + CRT. The samples made of mixtures FBC FA and FBC + CRT probably contained too much water in the system in relation to the water demand of ingredients. After 7 days of curing, samples could still contain an excess of water, which evaporated rapidly after the demolding of the samples. In the case of samples made of mixture FBC FA + M (25%), the shrinkage was visible in noticeably shorter dimensions during measuring samples using a caliper. The samples lost ~2% of their height, width, and length during the rest of the 21 days of curing. However, in the case of samples made of mixture FBC FA + M (25%) no visible cracks appeared on the surface (Figure 4a).

**Figure 5:**
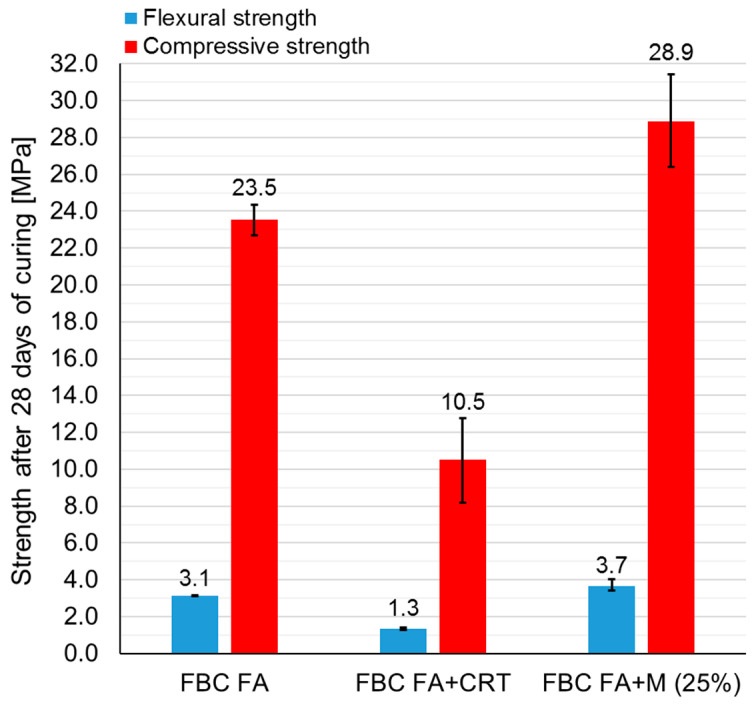
Flexural and compressive strength of geopolymer samples made of different mixtures (mean values for all series tested).

Figure 5 presents the results of flexural and compressive strength of samples made of mixtures FBC FA, FBC FA + CRT and FBC FA + M (25%), demolded after 7 days and tested after 28 days. The following number of samples were subjected to the test in each series: FBC FA and FBC FA + CRT, two samples subjected to flexural strength test and four samples subjected to compressive strength test, and FBC FA + M (25%), three samples subjected to flexural strength test and six samples subjected to a compressive strength test. Table 4 presents the determined values of standard deviations and coefficient of variations (CoV) of all results. The samples made of mixture FBC FA + M (25%) achieved considerably higher flexural and compressive strength than the rest of the samples. The lowest results were noticed in the case of samples containing CRT glass. Those samples were also characterized by the highest CoV values of compressive strength results. The relatively high strength of samples from series FBC FA indicated that the net of cracks covering the surface was rather shallow and did not affect the structure significantly.

Nevertheless, due to the appearance of the samples after 28 days of curing, mixtures FBC FA and FBC FA + CRT were not further considered during the next phase of the investigation. The mixture FBC FA + M (25%) was chosen for further tests. Samples made of this mixture achieved relatively high flexural and compressive strength after both 7 and 28 days of curing. In the next step of the investigation, the authors tried to find an optimal curing regime that could limit the samples’ shrinkage. Samples made of mixture FBC FA + M (25%) were characterized with the brittle failure mode visible in Figure 4b. The brittleness of samples resulted in some minor damages of edges (visible in Figure 4) caused during the demolding of samples with the use of compressed air. This feature of geopolymer should be considered during future investigations.

The densities of all samples discussed in Section 4.1 and Section 4.2 are presented in Table 5. The density of samples made of all mixtures decreased significantly (9–14%) during the period of curing without molds. Samples containing CRT glass were characterized with the highest density. The densities of samples from the other series did not differ significantly.

### 4.3. The Influence of Regime of Curing on Mechanical Behavior

**Figure 6:**
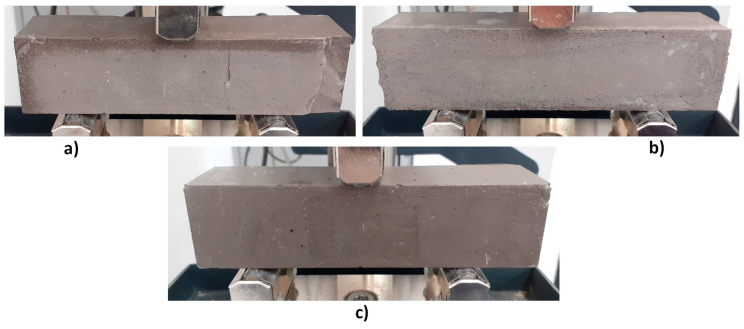
Ready to three-point bending test samples made of mixture FBC FA + M (25%) demolded after: (**a**) 7 days; (**b**) 14 days; (**c**) 28 days.

In this part of the investigation, the influence of the time of demolding of the samples on mechanical behavior was examined. Only samples made of mixture FBC FA + M (25%) were subjected to this part of the research. Samples were demolded after 7, 14 or 28 days of curing. All samples were tested after 28 days. Samples demolded after 7 and 14 days were kept in the laboratory at ambient temperature for the rest of the curing period. Each series consisted of three samples subjected to a flexural strength test and six samples subjected to a compressive strength test. Figure 6 presents the appearance of samples subjected to different curing regime just before the test.

**Figure 7:**
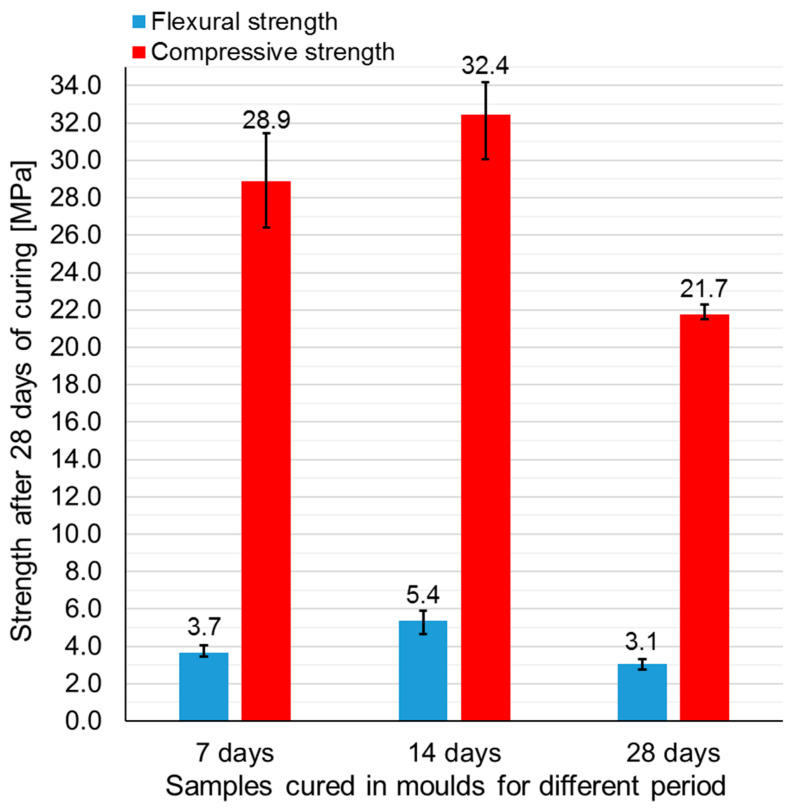
Flexural and compressive strength of geopolymer samples demolded after 7, 14, and 28 days of curing (mean values for all series tested).

Figure 7 presents all strength results. Table 6 contains the standard deviations and coefficient of variations. According to the graph, the time of demolding has a significant influence on mechanical performance. The highest flexural and compressive strength was achieved by samples demolded after 14 days of curing (5.4 and 32.4 MPa, respectively), while samples demolded after 28 days achieved the lowest strength values (3.1 and 21.7 MPa, respectively). The flexural strength of samples demolded after 28 days was lower than the flexural strength of samples demolded after 7 and 14 days, respectively by 16 and 43%. In case of compressive strength, the differences were equal to 25 and 33%, respectively. The lowest strength of samples demolded after 28 days could be caused by the excess of water contained inside the structure. The same conclusion can be drawn by comparison of the colour of the samples (Figure 6). As can be noticed, the surface of samples demolded after 7 and 14 days was brighter than the surface of samples demolded after 28 days. The difference in colour was probably caused by the fact that samples demolded after 28 days contained excess water, which had no time to escape. Those samples were demolded just before the strength test, therefore the excess of water had no time to evaporate in contrary to samples demolded after 7 and 14 days, which had contact with air for 21 and 14 remaining curing days, respectively. The average density of the samples (Table 7) also confirms this explanation. The density of samples demolded after 28 days was ~16% higher than that of samples demolded after 7 or 14 days. In turn, according to the results, 7 days transpired to be too short a period for the full geopolymerization of geopolymer samples based on FBC fly ash and cured at the ambient temperature. Therefore, samples demolded after 7 days achieved lower strength values than samples demolded after 14 days. According to Table 6, samples cured longer in molds achieved more stable compressive strength results (lower coefficient of variation). Concluding the presented results, the author decided that among all investigated curing conditions (curing in molds by 7, 14, and 28 days), curing for 14 days in molds was the most effective according to high mechanical performance. Summarizing the presented results, it can be assumed that among all the tested curing conditions (in the forms 7, 14, and 28 days), curing 14 days in the molds was the most effective due to the high mechanical parameters. The mechanical parameters after 10 and 20 days have not been tested, therefore the obtained maximum values may in fact occur for a hardening period slightly shorter or longer than 14 days, which requires confirmation in additional tests.

The results of the tests presented above are generally consistent with the results presented in other publications. However, according to the authors, this article brings some additional information to the existing knowledge on FBC ash geopolymers.

FBC FA + M (25%) containing FBC fly ash to metakaolin in a mass ratio of 3:1 was selected as the best mix in terms of possible application in the production of prefabricated building elements. The samples made of this mixture were characterized by the highest bending and compressive strengths among those tested after 7 and 28 days of hardening. The short time for the material to obtain sufficiently high strength is one of the basic parameters for determining its suitability for construction prefabrication. An additional advantage of this mixture is the low concentration of sodium hydroxide (6 mol/L), which was used together with sodium silicate for activation. A lower activator concentration is an economical and environmentally friendly solution. Deforming the geopolymer after 14 days leads to the best mechanical properties and limited shrinkage. Samples can be cured at ambient temperature, which is an ecological, low-labor and cheap solution.

## 5. Conclusions

The article presents the results of the first stage of research on determining the optimal composition and hardening regime of a geopolymer based on locally available fly ash from a circulating fluidized bed in the aspect of obtaining appropriate compressive strength and flexural strength. These two parameters are the basic ones that may decide on the possibility of its use in the production of prefabricated building elements. Of course, further research is necessary in order to better recognize this material, its features, and its properties, especially in terms of its long-term behavior, compatibility and cooperation (anchoring and bond strengths) with various types of reinforcement, including non-metallic reinforcement.

Nevertheless, based on the results obtained in the presented investigation, the following main conclusions can be drawn:

1. FBC fly ash used in the research can be used to produce a geopolymer with good mechanical properties. The average values of the maximum compressive and bending strength obtained during the tests were 32.4 and 5.4 MPa, respectively, which is sufficient for the material for construction prefabrication. Geopolymer with such parameters had a relatively low density, compared to classic gravel concrete, equal to 1450 kg/m^3^.
2. The presented studies showed that the addition of CRT glass is associated with a decrease in the compressive and flexural strength of the FBC FA geopolymer compared to the FBC FA geopolymer without any additives.
3. Of all the tested compositions of geopolymer, the optimal results were obtained for the geopolymer containing FBC to metakaolin in a weight ratio of 3:1 and activated with sodium silicate and sodium hydroxide at a concentration of 6 mol/L.
4. The addition of metakaolin in the range of 25% has a positive effect on the mechanical parameters of the FBC FA geopolymer. It caused an increase in bending strength by up to 267% and an increase in compressive strength by up to 78% compared to the studied geopolymer of other compositions.
5. Too early demolding of the geopolymer may lead to cracks and significant material shrinkage.
6. The best strength results (measured after 28 days) were achieved by samples cured in molds for the first 14 days. The entire hardening process took place at room temperature.

The achieved and described results show that proposed FBC fly ash-based geopolymer is a promising, light material of good mechanical characteristics. Tested geopolymer allows for utilization of a considerable amount of waste and does not need extra energy for curing at an elevated temperature. In the next stage of research, the authors intend to focus on a further, more detailed study and description of the presented geopolymer based on local FBC fly ash, especially in terms of its structure and the chemical reactions taking place in it. Of course, the long-term behavior of such a polymer and the changes in its properties over time, as well as the cooperation with the reinforcement (metallic or non-metallic) and the determination of the microstructure must also be carefully examined. In addition, the authors plan research on the optimal selection of the type and content of aggregate, and research on larger samples, including long-term studies.

## Figures and Tables

**Table 1:** Chemical composition of FBC fly ash ^1^, metakaolin ^2^ and CRT glass ^3^.

Component	Oxide Composition (wt %)		
	FBC Fly Ash	CRT Glass	Metakaolin
SiO_2_	44.20	76.10	53.12
Al_2_O_3_	21.20	1.37	42.14
CaO	15.63	5.24	0.44
Fe_2_O_3_	6.14	0.38	0.45
Na_2_O	0.92	6.25	0.09
BaO	0.15	2.62	-
K_2_O	2.15	2.36	0.73
MgO	2.13	1.64	0.26
PbO	-	1.61	-
SrO	-	1.42	-
SO_3_	5.05	0.55	-
ZrO_2_	-	0.28	-
TiO_2_	0.78	0.12	0.64
ZnO	-	0.05	-
As_2_O_3_	-	0.01	-
H_2_O	-	-	0.22
P_2_O_5_	0.47	-	0.03
Cl	-	-	0.02
S	-	-	0.01
MnO	0.068	-	0.01
C	1.11	-	-

^1^ Data obtained from the supplier Tauron Polska Energia^®^. ^2^ Data obtained from the producer: Astra Technologia Betonu^®^. ^3^ Chemical composition was determined by the XRF analysis done by EkotechLAB^®^.

**Table 2:** Mixture composition.

Mixture	FBC FA [kg/m^3^]	CRT Glass [kg/m^3^]	Metakaolin [kg/m^3^]	Sodium Silicate [kg/m^3^]	NaOH [kg/m^3^]	Si/Al [-]	Na/Al [-]
FBC FA	922	0	0	622	310 (10M)	2.51	1.29
FBC FA + CRT	606	606	0	476	240 (10M)	2.64 **^1^**	1.50 **^1^**
FBC FA + M (25%)	692	0	230	513	303 (6M)	1.98	0.77
FBC FA + M (20%)	737	0	184	716	208 (10M)	2.25	0.97

^1^ Ratios calculated for FBC fly ash, sodium silicate and sodium hydroxide (without CRT glass).

**Table 3:** Standard deviation and coefficient of variation of series of samples tested after 7 days.

Mechanical Property	Standard Deviation [-] (CoV in [%])			
	FBC FA	FBC FA + CRT	FBC FA + M (25%)	FBC FA + M (20%)
Flexural strength	0.06 (7%)	0.06 (5%)	0.20 (9%)	0.09 (15%)
Compressive strength	0.41 (3%)	0.36 (4%)	0.84 (5%)	0.46 (3%)

**Table 4:** Standard deviation and coefficient of variation of series of samples tested after 28 days.

Mechanical Property	Standard Deviation [-] (CoV in [%])		
	FBC FA	FBC FA + CRT	FBC FA + M (25%)
Flexural strength	0.04 (1%)	0.09 (6%)	0.33 (9%)
Compressive strength	0.72 (3%)	1.99 (19%)	2.10 (7%)

**Table 5:** Density of samples made of different mixtures, measured after 7 and 28 days of curing.

Curing Period	Density [kg/m^3^]			
	FBC FA	FBC FA + CRT	FBC FA + M (25%)	FBC FA + M (20%)
7 days	1740	1890	1750	1750
28 days	1540	1720	1500	X

**Table 6:** Standard deviation and coefficient of variation of series of samples tested after 28 days and demolded after 7, 14, and 28 days.

Mechanical Property	Standard Deviation [-] (CoV in [%])		
	7 Days	14 Days	28 Days
Flexural strength	0.33 (9%)	0.65 (12%)	0.27 (9%)
Compressive strength	2.10 (7%)	1.77 (5%)	0.33 (2%)

**Table 7:** Density of samples demolded after 7, 14 or 28 days, measured after 28 days of curing.

Density [kg/m^3^]		
7 days	14 days	28 days
1500	1450	1710

## Data Availability

Data available on request.
